# Next Generation Bone Marrow Adiposity Researchers: Report From the 1^st^ BMAS Summer School 2021

**DOI:** 10.3389/fendo.2022.879588

**Published:** 2022-04-13

**Authors:** Rossella Labella, Sarah Little-Letsinger, Viktorjia Avilkina, Rita Sarkis, Michaela Tencerova, Annegreet Vlug, Biagio Palmisano

**Affiliations:** ^1^ Department of Physiology and Cellular Biophysics, Columbia University Medical Center, New York, NY, United States; ^2^ Department of Evolutionary Anthropology, Duke University, Durham, NC, United States; ^3^ Marrow Adiposity and Bone Lab (MAB Lab) ULR4490, Univ Littoral Côte d’Opale, Boulogne-sur-Mer, France; ^4^ Laboratory of Regenerative Hematopoiesis, Institute of Bioengineering, Ecole Polytechnique Fédérale de Lausanne, Lausanne, Switzerland; ^5^ Department of Biomedical Sciences, University of Lausanne, Lausanne, Switzerland; ^6^ Molecular Physiology of Bone, Institute of Physiology of the Czech Academy of Sciences, Prague 4, Czechia; ^7^ Section of Endocrinology, Department of Internal Medicine, Center for Bone Quality, Leiden University Medical Center, Leiden, Netherlands; ^8^ Department of Molecular Medicine, Sapienza University of Rome, Rome, Italy

**Keywords:** bone marrow adipose tissue (BMAT), bone marrow adiposity, bone marrow adipocytes, metabolism, imaging technique, BMSC – bone marrow stromal cells, histology, career development

## Abstract

The first International Summer School on Bone Marrow Adiposity was organized by members of Bone Marrow Adiposity Society and held virtually on September 6-8 2021. The goal of this meeting was to bring together young scientists interested in learning about bone marrow adipose tissue biology and pathology. Fifty-two researchers from different backgrounds and fields, ranging from bone physiopathology to adipose tissue biology and hematology, participated in the summer school. The meeting featured three keynote lectures on the fundamentals of bone marrow adiposity, three scientific workshops on technical considerations in studying bone marrow adiposity, and six motivational and career development lectures, spanning from scientific writing to academic career progression. Moreover, twenty-one participants presented their work in the form of posters. In this report we highlight key moments and lessons learned from the event.

## Background

**Figure 1:**
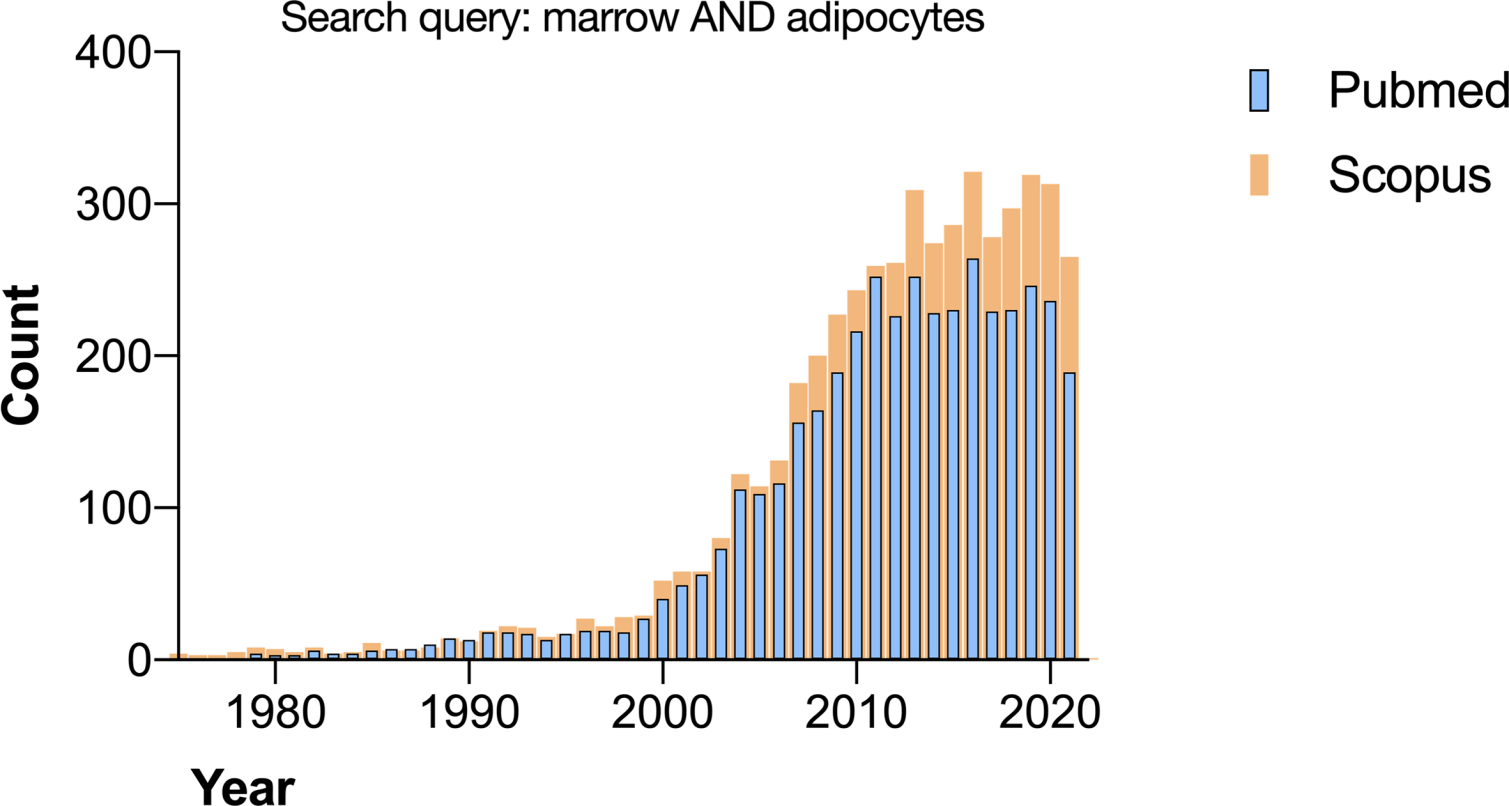
Search results for “marrow AND adipocytes” on Pubmed and Scopus performed in November 2021.

The growing interest in bone marrow adipose tissue (BMAT) is evident from the increase in articles published yearly by researchers all over the world (**Figure 1**). The credit for bringing this field to international attention is given to Dr. Mehdi Tavassoli with his works in the early 70’s (1), although observations of the transformation of red marrow into yellow marrow have been published since the 1800’s (2, 3). In the last twenty years, the study of BMAT has emerged as an important field in bone and bone marrow biology.

Beginning in 2015, international meetings on Bone Marrow Adiposity (BMA) have been organized with the aim to bring together scientists working on or interested in BMAT (4–8). Later in 2017, a group of BMA scientists founded the BMA Society (BMAS) (http://bma-society.org/) with Dr. Pierre Hardouin as the founding president. The aims of the newly established society were to advance the knowledge of BMA by facilitating interdisciplinary exchanges, developing research strategies, and standardizing nomenclature and methods to study BMAT. Thus far, the society has produced and published three consensus articles (9–11).

**Figure 2:**
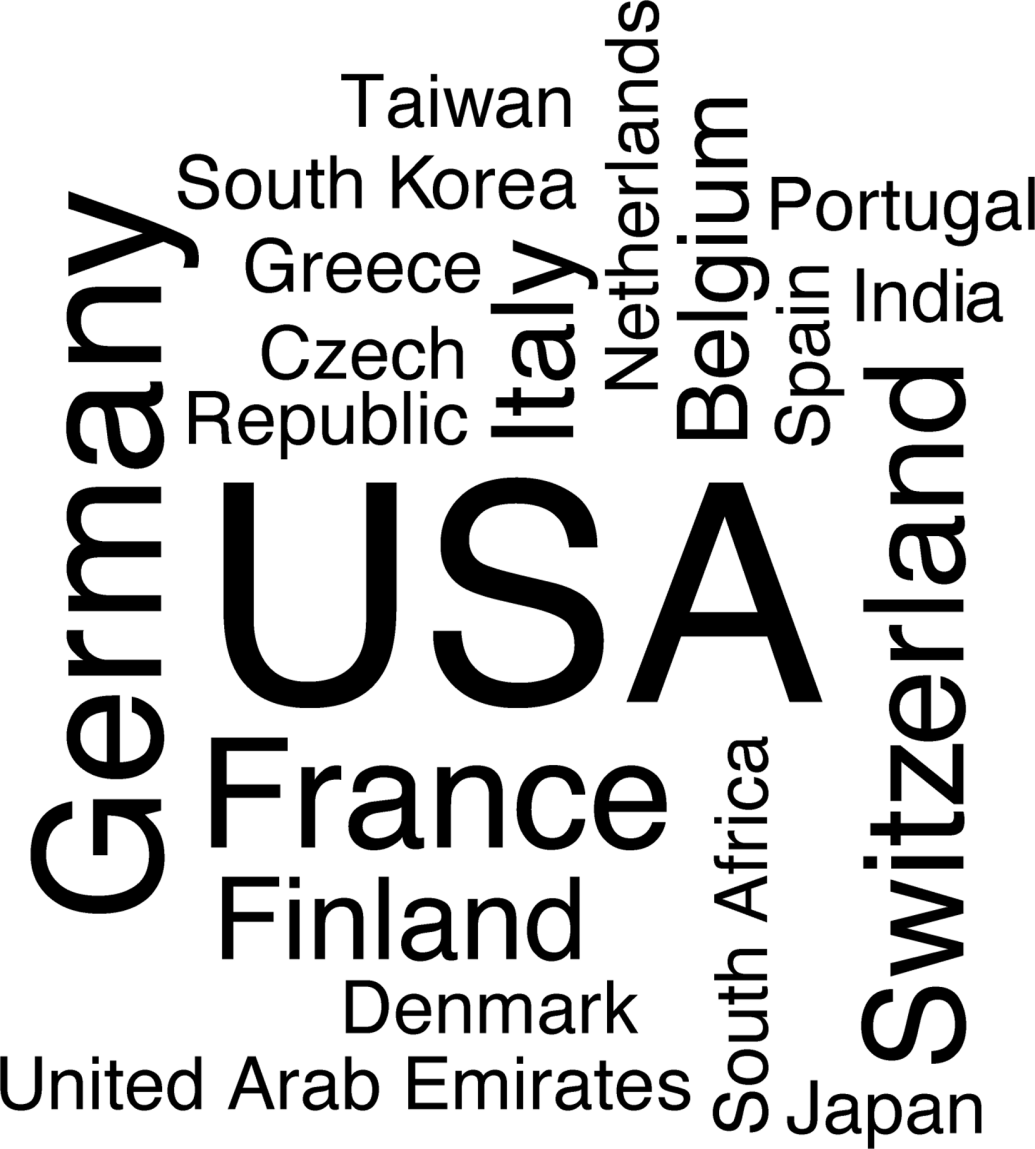
Word cloud performed with the countries of origin of the attendees at the 1^st^ BMAS Summer School. The number of attendees from each country correlates with the word size in the cloud. The higher the number of attendees, the bigger the word. The analysis was carried out with WordCloud package in R.

One of BMAS’s key goals is to promote BMAT research by training and supporting young scientists interested in this field. Therefore, a group of young scientists from Europe and the United States envisioned the first BMAS Summer School, which was held as a virtual meeting on September 6-8, 2021. The goal of the event was to foster general knowledge of BMAT research to young researchers from all fields in order to provide fundamentals about how BMAT originates, develops, and interacts with other organs, as well as how to study BMA and what methods to use in both clinical and basic research. Moreover, the event program contained career development workshops with the aim to provide tips on how to face difficulties and overcome obstacles in the academic life, which alternative paths exist outside academia, and how to write a good manuscript and how to understand where to publish. Fifty-two participants from all over the world (**Figure 2**) attended the meeting, with active participation, live discussion, and incredible enthusiasm.

The three-day event consisted of a daily keynote lecture on BMA, followed by a scientific workshop on how to study BMA and two motivational and career development workshops with a total of twelve presentations from invited speakers. Moreover, twenty-one abstracts were selected and presented as posters with a live discussion; these abstracts have been published and are available elsewhere (12).

## Lectures on Bone Marrow Adiposity

### BMAT: Origin, Multicellularity, Regulation of Lineage Commitment, and Functional Implications

Dr. Moustapha Kassem (University of Southern Denmark) opened the three-day event with a lecture entitled “BMAT: origin, multicellularity, regulation of lineage commitment, and functional implications”. In his talk, Dr. Kassem described a historical roadmap from the first correlations with bone diseases, such as osteoporosis, to the most recent studies on the regulation of bone marrow stromal cell differentiation potential towards osteoblasts or bone marrow adipocytes. This session was moderated by Dr. Gustavo Duque (University of Melbourne). Dr. Kassem gave an overview of the major findings and discoveries on BMA in relation to bone health and homeostasis and its relevance to clinical outcomes. His talk pointed out the concept of cellular heterogeneity of bone marrow stromal cells (BMSCs) and presence of committed adipogenic and osteogenic progenitors besides multipotent BMSCs, which contribute to the interaction between bone and fat mass and cellular changes in the bone marrow microenvironment (13). In order to understand the mechanisms underlying age- and osteoporosis-related changes in bone formation, his laboratory discovered several secreted factors derived from BMSCs [e.g., Delta Like Non-Canonical Notch Ligand 1(DLK1), Legumain (LGMN), Cell Migration Inducing Hyaluronidase 1(KIAA1199)], which play a key role in stem cell differentiation and provide a link between bone biology and whole body energy metabolism (14–17). At the end of his talk, Dr. Kassem presented data on human BMSCs isolated from obese subjects demonstrating a hypermetabolic and accelerated senescent phenotype in comparison to lean-derived BMSCs. Dr. Kassem’s work provides important insights into metabolic changes occurring in the bone marrow microenvironment and its relation to higher bone fragility in metabolic diseases (18, 19). These data will contribute to the explanation of the obesity paradox in bone biology, as obese subjects are characterized with unchanged or increased bone mass but high rates of bone fragility.

### BMAT and Bone/Bone Marrow Diseases

The second keynote lecture was given by Dr. Mara Riminucci (Sapienza University of Rome) and moderated by Dr. Ling Qin (University of Pennsylvania). The main focus of this talk was the role played by BMAT in bone and disease. She started by defining bone marrow adipocytes (BMAds), referring to Tavassoli’s definition, as recognized large cells with a single lipid droplet (20) and positive for markers such as Perilipin-1 and Adiponectin. She emphasized that both morphology and marker expression are crucial to recognizing BMAds. In terms of origin, BMAds appear in perinatal/post-natal life. The development of the “yellow marrow” (to distinguish from the hematopoietic “red marrow”) is a complex phenomenon tightly regulated by multiple genetic, developmental, and functional factors (2). *Ex vivo* transplantation studies have revealed a common progenitor for both BMAds and osteogenic cells that resides in the bone marrow stromal compartment (21–24). The fate choice of this common progenitor plays a crucial role in bone physiology and health. Functionally, one of the roles of BMAT is to fill the marrow space: after birth in fact, hematopoietic bone marrow becomes progressively filled with adipocytes among hematopoietic cells. However, BMA is highly flexible and reversible. Besides the space filling function, BMAT contributes to systemic metabolism (25), supports hematopoiesis (26), and modulates cell differentiation within the bone marrow stroma (27). Moreover, based on their localization as perivascular cells, BMAds regulate the sinusoid caliber and distribution of marrow blood flow (28).

The second part of the lecture focused on the role of BMAT in the context of human pathology. BMAT is, in fact, involved in different pathologies of the bone and bone marrow, including osteoporosis, bone fragility, hematological tumors, bone cancer metastasis, and some genetic disorders involving the skeleton (29, 30). On the other hand, BMAT may have its own tissue-specific pathology such as lipoma and hibernoma (31, 32).

### Metabolic Function of Bone Marrow Adipose Tissue

The third keynote lecture was provided by Dr. William Cawthorn (University of Edinburgh) and moderated by Dr. William Ferris (Stellenbosch University). The presentation focused mainly on BMAT’s impact on metabolic and skeletal homeostasis within bone marrow. Dr. Cawthorn began his lecture by introducing subtypes of adipose tissue, their location, and structure. As extensive research has been done to characterize white adipose tissue (WAT) and brown adipose tissue (BAT), the talk focused on their metabolic functions, covering the processes of energy storage and energy release within WAT, as well as thermogenesis in BAT. In contrast with WAT and BAT, little is known about BMAT metabolic characteristics and its systemic function. As a leading expert in the study of BMAT, Dr Cawthorn, presented a detailed description of lipid content properties and metabolism (e.g., lipogenesis and lipolysis) in BMAT from different skeletal sites. Furthermore, his research group conducted transcriptional characterization comparing BMAT and WAT using microarray analysis. The results demonstrated that BMAT is less responsive to insulin compared to WAT (33). These findings were tested by PET/CT scan, which showed a lack of insulin-stimulated glucose uptake in BMAT (distal tibia), concluding that BMAT is insulin resistant (33). Additionally, Dr Cawthorn’s group demonstrated that BMAT is not cold responsive, as it does not increase glucose uptake during cold exposure (33, 34). Another finding, suggested that BMAT glucose uptake is able to influence systemic glucose homeostasis (33, 35). This lecture concluded with the statement that BMAT is a metabolically distinct fat depot with importance in numerous clinical conditions.

## Workshops on Bone Marrow Adiposity

### Standard Nomenclature, Abbreviation, and Units for the Analysis of Bone Marrow Adiposity

The first workshop entitled “Standard nomenclature, abbreviations and units for the analysis of bone marrow adipose tissue” was given by Dr. Nathalie Bravenboer (University Medical Center) and moderated by Dr. Stefanie Lucas (Université du Littoral Côte d’Opale). Dr. Bravenboer started with some historical data of the discovery of the bone marrow space and role in red blood cell development (36). The first part of her talk can be summarized with the mission of the nomenclature working group: “Our purpose is not to encourage or discourage the use of abbreviations and symbols but to ensure that the same ones are used by everybody” (10). Dr. Bravenboer listed some consensus notes: first, BMAd is the adopted abbreviation for the bone marrow adipocytes; second, BMA can be considered as a tissue, and third, anatomical location is important to give reference due to documented differences in sub-depots (e.g., proximal versus distal in the mouse tibia). The last part of this workshop was dedicated to a description of the methodologies for the analysis of BMAT including histomorphometry, Magnetic Resonance Imaging (MRI), Magnetic Resonance Spectroscopy (MRS), and Computed Tomography (CT) analysis, along with a full list of the parameters adopted to define BMAT while using each of those techniques. A detailed list of all the nomenclature and methodologies recommendations can be found in the two consensus papers published in 2021 (9, 10).

### Skill Development on Histology

The second skill development workshop featured an invited talk by Dr. Erica Scheller (Washington University in St. Louis) covering histology of BMAT and was moderated by Dr. Michaela Tencerova (Institute of Physiology of the Czech Academy of Sciences). Dr. Scheller began the session outlining the potential uses of histology to study BMAds, including cell number, size, localization, molecular analyses, and lineage tracing, with a focus on the importance of technical considerations decided *a priori*. While the necessary considerations vary across specific histologic techniques, some factors of notable importance were generally applicable. Consistency in region of interest (ROI), analysis of a large and representative ROI, sufficient sample size (*e.g.*, 100-300 adipocytes per sample), and the use of controls are critical to successful and reproducible histology. Hematoxylin and eosin staining of formalin-fixed paraffin-embedded tissue is ideal for investigating cell number and size (9, 37, 38). Investigation of localization or co-localization requires the usage of immunostaining methods, including both immunohistochemistry, utilizing a single antibody, and immunofluorescence, enabling multi-antibody staining (39, 40). Antibodies must be validated and optimized through positive and negative controls, including omission of the secondary antibody and tissue from genetic knockouts (41). Molecular analyses and BMAd-specific protein expression can be difficult to assess using immunostaining alone and may require lineage tracing or reporter lines (42). Despite its limitations, histology is a powerful tool for the study of BMAds and can be highly informative when care is taken to ensure rigor and reproducibility through the employment of appropriate controls and technical considerations. In summary, this workshop was an excellent introduction to the histology of BMAds for students and researchers new to the technique and will enable the usage of histological best practices.

### Standard Imaging Methods in the Assessment of Bone Marrow Adipose Tissue

Dr. Dimitrios Karampinos (Technical University of Munich) delivered the third BMAT workshop: “Standard imaging methods in the assessment of bone marrow adipose tissue” that was moderated by Dr. Greet Kerckhofs (Katholieke Universiteit Leuven). Dr. Karampinos provided an extensive overview of the imaging techniques in use to assess both BMAT and bone properties, outlining the difficulties to explore both with the same imaging technique. He explained the concept of Magnetic Resonance (MR) contrast mechanisms to study fat, the T1-weighted imaging and chemical shift encoding resulting in a proton density fat fraction (PDFF). The PDFF has been used to demonstrate the fat fraction increases with aging (43, 44) and is higher in patients with osteoporosis (45). To explore in more depth the composition of fatty acids in BMAT, unsaturated and saturated fat can be measured. Data shows that saturated fats are lower in patients with fractures and/or diabetes (46), although this remains a challenging measurement especially in regions with more hematopoietic marrow (47). Finally, the newest development in BMAT imaging, i.e. measuring lipid droplet size using MRI techniques, was discussed (48). This talk was extremely useful to improve the understanding of *in vivo* measurement of BMAT.

## Career Development Workshops

### From Scientific Writing to Getting Your Paper Published

The first Career Development Workshop was given by Dr. Jonathan Tobias (University of Bristol) and moderated by Dr. Claire Edwards (University of Oxford). As a Specialty Chief Editor of the Bone Research Section in Frontiers in Endocrinology, Dr. Tobias focused on aspects of writing a scientific paper and going through the publication process. This talk covered the basic paper and journal types, reviewed the paper preparation process (e.g., paper structure and content), and the process of choosing appropriate journals for submission. Moreover, Dr. Tobias discussed the peer review process, covered the types of peer review and emphasized the importance of this process to improve the quality of publication. He also provided guidelines for responding to reviewers and tips on “Surviving the Peer Review” process, which was highly useful and informative for young researchers who are currently undergoing these processes with their first publications.

### Academic Career Progression

The academic career progression talk was given by Dr. Michaela Reagan (Maine Medical Center Research/Tufts University and was moderated by Dr. Annegreet Veldhuis (Leiden University Medical Center). During the Academic Career Progression workshop, Dr. Reagan first took us with her on her academic journey and highlighted the importance of giving talks and meeting people during conferences to obtain new positions and advance your career. She shared her motivation to pursue an academic career and her keys to success, including diversification, finding a good mentor, considering work life balance, and personal traits like resiliency, open-mindedness, and most importantly, enjoying your work! Common mistakes were also discussed, such as being too optimistic, perfectionism, never saying no, and imposter syndrome. She explained the different academic career paths (tenure track, non- tenure track, or adjunct faculty) and highlighted useful grant options in the USA (e.g., NIH F31, F99/K00, NIH F32, DoD Postdoc Fellowship, or other Foundations and society). All in all, this workshop helped to gain more insight into the academic career progression and get some very useful advice.

### How I Write a Grant

The career development workshop titled “How I write a grant” was given by Dr. Christopher Hernandez (Cornell University) and moderated by Dr. Julien Paccou (Lille University Hospital). Dr. Hernandez began the session detailing the importance of writing clarity in not only increasing the chances of being awarded the grant, but also in receiving feedback that will make the next grant better in the event it is not awarded. To this end, providing a strong framework, directly stating answers to review criteria, and using definitive verbiage (e.g., determine, establish, etc.) in your specific aims can improve the likelihood of receiving funding. Another key aspect of writing a grant is striking a balance between the big picture and technical details. Consideration of varying backgrounds of potential reviewers and the circumstances the grant will be read in (e.g., after hours, weekends), as well as developing skills in science communication can be extremely helpful. Numerous resources are available for additional help, including seminars, workshops, and published workbooks (49).

### How to Start a Collaboration

This career development workshop continued with an invited talk by Dr. Stavroula Kousteni (Columbia University Irving Medical Center) on how to start a collaboration and was moderated by Dr. Bram van der Eerden (Erasmus University Rotterdam). This workshop was led in a very interactive format. Dr. Kousteni began by directly asking the participants what are the main aspects/points that you look for in a new collaborator (e.g., expertise, new methodology, clinical samples, animal models) as it can help to refine your search for the best collaborator. She also mentioned it is important to set up the rules of the collaboration at the beginning in order to avoid any miscommunication and delay progress of the project. Another trick to establishing successful collaborations is to find a collaborator with a similar personality. In addition, this workshop summarized several positive and negative aspects of starting new collaborations and left us with several tips on what is crucial in this important part of our scientific work.

### A Path to the Science of the HeART

The career development talk entitled “A path to the science of the HeART” was given by Dr. Ana Silva and was moderated by Dr. Rossella Labella (Columbia University). Dr. Silva shared her personal transition from postdoc in academia to a freelancer position as a scientific illustrator. Like the majority of early-stage investigators in academia, she was considering becoming a Principal Investigator as her first choice. She was excluding other career options for three main reasons: ego, expectation, and fear. With the help of the MIND (Making Informed Career Decisions) program at the Gladstone Institute she decided to mix her two passions, science and illustration, and to pursue an alternative career path as a scientific illustrator. This led to the opening of her own business in Portugal. She concluded the workshop with several pros and cons of being a freelancer worker.

### Learning from Failure in Life and Research

The last talk of the BMAS Summer School 2021, was given by Dr. Jennifer Heemstra (Emory University) and moderated by Dr. Erica Scheller (Washington University in St. Louis). The main goal of this talk was to share the tremendous lessons that Dr. Heemstra has learned from her scientific and life experiences. She first invited the audience to reflect on their own challenges and lessons in their career path. She began with a first lesson: your success is determined by what you do when everything is going wrong; and added that our life is the most interesting experiment that we get to run. She reminded the audience that there will be times in research and in life where things do not go as planned. It is true that these challenges and failures are frustrating, but they can also be an opportunity for us to learn and grow. She shared the many challenges she has faced in her career, including being advised not to pursue a scientific career by one of her high school academic advisors, her participation in Science Olympiad, challenging herself with the organic course chemistry where she discovered her passion for chemistry, losing her father and one of her best friends, becoming pregnant during her first week of postdoctoral training, and not passing the tenure votes. Dr. Heemstra reflected on not receiving tenure as one of the worst times of her life because she felt that she disappointed both her family and lab members. Through all of these challenges she discovered that she is stronger than she thought.

Finally, we should learn to enjoy what we are doing and do it with passion. Discovering the passion is essential and fulfilling. However, we should develop our passion rather than follow it. Dr. Heemstra ended the talk by encouraging the audience to take a step out of their comfort zone every day to challenge our capabilities. One lesson we learned from this talk: what might be your biggest failure can empower you to the most life-changing experience in your whole life.

### Poster Sessions

**Figure 3:**
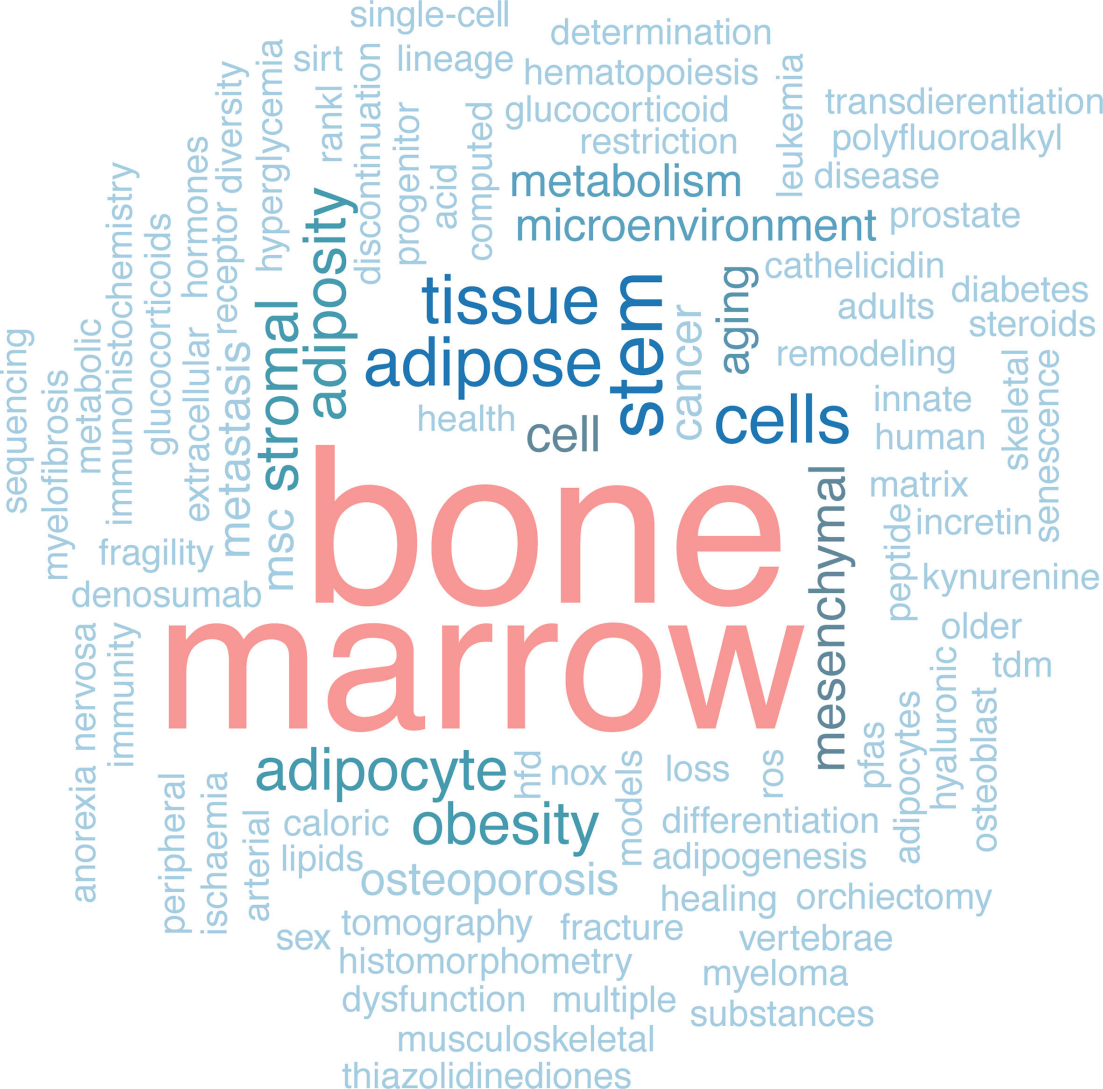
Word cloud of the abstract keywords. The keyword size correlates with the number of times that keyword was used in the abstracts. The analysis was carried out with WordCloud package in R.

Poster sessions consisted of a combination of pre-recorded 3-minute video presentations and a live discussion over Zoom in individual rooms. Abstracts covered a wide range of topics (**Figure 3**) and were divided into three categories, one per day of the summer school: 1) BMAT and stem cells, 2) BMAT, aging and cancer, and 3) BMAT and metabolic diseases. A detailed list of poster presenters and abstract titles is available in **Table 1**, while abstracts with consent for publication are available online (12).

**Table 1:** List of the presenters and titles of the three poster sessions.

Presenter	Poster Title
**1) BMAT and stem cells**	
Viktorija Avilkina	How the severity level and duration of energy deficit in mice model affects the bone phenotype, bone marrow adiposity and bone marrow stromal cells differentiation capacity?
Federica Quacquarelli	Ex vivo human cellular models to study adipocyte-induced trans differentiation of osteoblasts.
Drenka Trivanović	Human bone marrow adipose tissue niche supports mesenchymal progenitors with unique metabolic and stem cell features.
Amélie Paquet	Bone Marrow Adipocytes differentiated *in vitro* express the mRNA of the human cathelicidin antimicrobial peptide (hCAMP).
Thomas H. Ambrosi	Distinct lineage fates of diverse skeletal stem cell types orchestrate long bone physiology.
George Soultoukis	Physiological And Nutrition-related Effects on Bone Marrow Adipocyte Formation.
Vagelis Rinotas	Investigating the effects of Denosumab treatment and discontinuation in bone marrow adiposity through analysis of Tg/RANKL osteoporotic mice
**2) BMAT, aging and cancer**	
Laura Trainor	Bone marrow adipocyte phenotype is altered early in progression to multiple myeloma.
Stefan Ruschke	Variation of the human vertebral bone marrow water T2 relaxation time assessed by *in vivo* single-voxel magnetic resonance spectroscopy.
Michele Dello Spedale Venti	Morphologic and phenotypic changes of human bone marrow adipocytes in marrow metastasis and chronic myeloid neoplasia.
Charlotte Rinne	The Effects of Nutrients on Stem Cell Function and Regeneration in Bone Tissue in Response to Ectopic Adipogenesis.
Wangjie Liu	Deciphering the hierarchy of human bone marrow stromal cells.
Laimar C Garmo	Effects of Per- and Polyfluoroalkyl Substances on Bone Marrow Adipose Environment: Potential Implications for Bone Metastatic Cancers.
Katelyn Greene	Assessing Bone Marrow Adipose Tissue in Older Adult Lumbar Vertebrae.
**3) BMAT and metabolic diseases**	
Husam Bensreti	Orchiectomy sensitizes the skeleton of male mice to the deleterious effects of kynurenine.
Dalia Ali	High Fat Diet (HFD)-Induced Obesity Augments the Deleterious Effects of Estrogen Deficiency in Bone. Evidence from ovariectomized Mice.
Rebecca L. Schill	Glucocorticoid Receptor in Bone Marrow Adipocytes is not Required for Expansion During Calorie Restriction.
Katja Wegener	Hyaluronan matrix in bone marrow adipose tissue: implications for the development and progression of insulin resistance.
Andrea Benova	The effect of novel thiazolidinedione analog on bone and metabolic parameters in animal model of diet induced obesity.
Jacob M. Bond	The role of Nox4-ROS in driving obesogenic bone marrow mesenchymal stem cell phenotype in mice.
*Not shown*	*Poster 14*

### Awards

All the abstracts were evaluated from the scientific board of the BMAS in a blinded mode, each abstract was evaluated by three scientific board members and the final score was the sum of the three different evaluations. For abstract evaluation, the following criteria were considered: effectiveness of background and hypothesis, novelty of the data, clearness of communication, and overall quality of writing. The abstract that got the highest score was entitled *Glucocorticoid receptor in bone marrow adipocytes is not required for expansion during calorie restriction* and presented by Dr. Rebecca L. Schill from University of Michigan, USA.

In addition, the organizing committee of the summer school gave awards for the best poster presentation and for the most active participant. The former was assigned to Laura Trainor from Medical Research Institute, Adelaide, Australia and the latter to Husam Bensreti from Augusta University, Georgia, USA.

## Concluding Remarks and Perspectives

Our understanding of BMA’s role and function in health and disease has grown considerably in the last years. Since 2015, an annual meeting on BMA has been organized with the aim to help collaborative research in the field and promote knowledge, which was accomplished with growing number of BMAS members and experts. In 2021, BMAS launched the first edition of the BMAS Summer School, an event organized by young scientists and addressed to trainees and junior investigators interested in the BMA field. Many biological questions remain to be addressed about the origin, development, and physiopathology of BMA, supporting the need to raise a young generation of scientists to the study of BMA. For instance, do BMAds share a common progenitor with osteoblasts? Why does BMAT develop? Do BMAds have only a filling function in bone marrow? Why do BMAds increase in conditions such as osteoporosis? Why do BMAds decrease with metastatic invasion of the bone marrow? Are there transcription factors and/or cell surface markers that are specific to BMAds and not other adipocytes? Will we be able to routinely measure BMAT in patients? Will it be possible in future to use BMAT assessment as diagnostic or prognostic tool?

BMAS Summer School 2021 was a success not only for the productive discussion coming from the insightful lectures of the speakers and the vibrant community of attendees, but also for bringing in scientists from fields other than BMA, and for the creation of a young investigator working group, the Next Generation BMAS. The main aim of the group will be to organize the biannual summer school, and other events like virtual happy hours, data discussion series, and “meet the professor” events, that will be included in the BMA meetings.

## Author Contributions

RL and BP served as co-chairs of the BMAS SS 2021 meeting, and S-LL, VA, RS, MT, and AV as members of the organizing committee. BP collected and analyzed metadata. RL, S-LL, VA, RS, MT, AV, and BP wrote and revised the manuscript. All authors approved the submitted version.

## Conflict of Interest

The authors declare that the research was conducted in the absence of any commercial or financial relationships that could be construed as a potential conflict of interest.

## Publisher’s Note

All claims expressed in this article are solely those of the authors and do not necessarily represent those of their affiliated organizations, or those of the publisher, the editors and the reviewers. Any product that may be evaluated in this article, or claim that may be made by its manufacturer, is not guaranteed or endorsed by the publisher.
